# Fabella Syndrome and Common Peroneal Neuropathy following Total Knee Arthroplasty

**DOI:** 10.1155/2021/7621844

**Published:** 2021-09-02

**Authors:** Connor C. Diaz, Avinesh Agarwalla, Brian Forsythe

**Affiliations:** ^1^Midwest Orthopaedics at Rush University Medical Center in Chicago, IL, USA; ^2^Department of Orthopaedic Surgery, Westchester Medical Center, Valhalla NY, USA

## Abstract

**Case:**

A 62-year-old man presented with persistent lateral knee pain 15 months following an uncomplicated total knee arthroplasty. There was a tendinous snapping structure over the lateral aspect of the knee in deep flexion with positive Tinel's sign over the fibular head. The patient underwent an uncomplicated flabella excision. The patient was cleared to return to work and full duty at two months postoperatively.

**Conclusion:**

Flabella syndrome is a rare but increasingly common mechanism of persistent lateral knee pain following total knee arthroplasty. Surgeons should be aware of this etiology of persistent lateral knee pain and offer treatment modalities to address this pathology.

## 1. Introduction

The fabella is a sesamoid bone in the lateral gastrocnemius that is present in 10-30% of the population [1–3]. Despite its high prevalence, the fabella rarely causes pathology leading to delayed diagnoses and prolonged patient discomfort when symptomatic [4]. Patients may develop fabella syndrome, which typically manifests insidiously as posterolateral knee pain during knee extension due compression along the lateral femoral condyle [5]. The fabella syndrome is often associated with a snapping or clicking sensation that is exacerbated by aerobic activates. In rare cases, the fabella has been reported to cause common peroneal neuropathy resulting in pain radiating to the anterolateral leg [6, 7]. When conservative interventions fail, surgical excision for treatment of fabella syndrome or fabella-associated common peroneal neuropathy has improved patient reported outcomes and returns to preinjury level of activities [5, 8].

## 2. Case Report

A 62-year-old man presented with persistent left lateral knee pain 15 months after total knee arthroplasty (TKA). He tolerated TKA well without intraoperative complications and made strong rehabilitation progress. The patient began experiencing a painful, snapping sensation along the posterolateral aspect of his left knee 1 month after TKA. Shortly after, he reported an associated shooting pain radiating from the lateral knee to his great toe and muscle spasms in his calf that interfered with ambulation. The patient received no relief from distal iliotibial band corticosteroid injection and physical therapy prior to presentation. His previous work-up included an electromyography, which demonstrated no evidence of left peroneal neuropathy, lumbar radiculopathy, plexopathy, sciatic neuropathy, or peroneal nerve lesion.

Inspection of the left knee showed mild effusion and a well-healed surgical scar. His left knee range of motion was 0° to 130°. No laxity with varus or valgus stress was appreciated. A snapping tendinous structure was palpated over the fibular head in deep flexion. The patient had positive Tinel's sign with palpation over the common peroneal nerve, posterior to the biceps femoris insertion.

Radiographic imaging showed a left total knee arthroplasty in good position without loosening, lysis, or fracture. A fabella was noted in the posterior aspect of the knee on the lateral view. Due to persistent discomfort, the patient elected for an open peroneal nerve neurolysis and ossicle excision of fabella.

## 3. Surgical Technique and Findings

**Figure 1:**
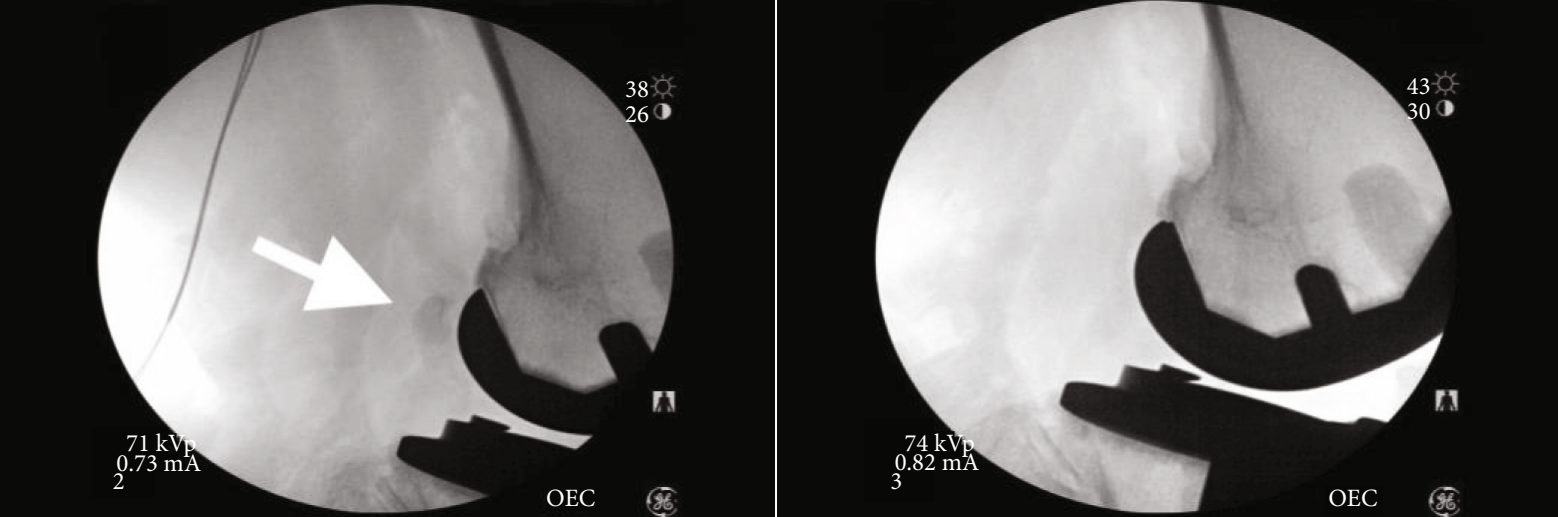
Intraoperative fluoroscopy. (a) Fabella before excision. (b) Knee after excision.

**Figure 2:**
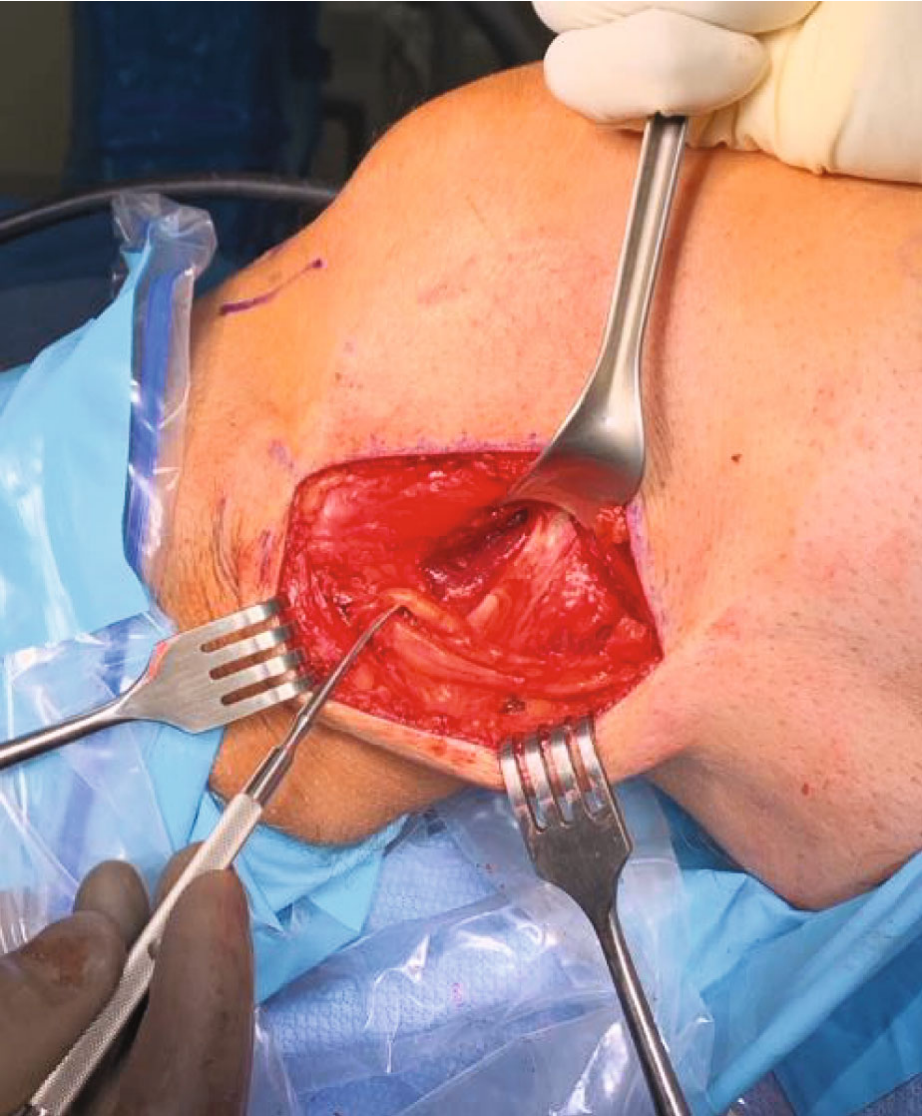
Bifid common peroneal nerve.

The procedure began with arthroscopic evaluation of the left knee, specifically the lateral femoral condyle and lateral gastrocnemius origin. Fluoroscopy was utilized intraoperatively to confirm fabella location (Figure 1). A 12 cm hockey stick incision was made from the lateral aspect of the knee, anterior to the fibula, and parallel to the posterior border of the iliotibial band. After careful dissection, the inferior iliotibial band was cut along its fibers to expose the short head of the biceps femoris. Next, tenotomy scissors were used to identify the peroneal nerve which emanated from the short head biceps femoris tendon. The peroneal sheath was released distally, where an additional branch of the peroneal nerve was identified, consistent with a bifid structure (Figure 2). A fibrosis tissues loop, tethering the bifid peroneal nerve together, was removed. The neurolysis release continued distally until just proximal to the fibular neck, yielding improved excursion of the peroneal nerve without constricting fiscal bands.

**Figure 3:**
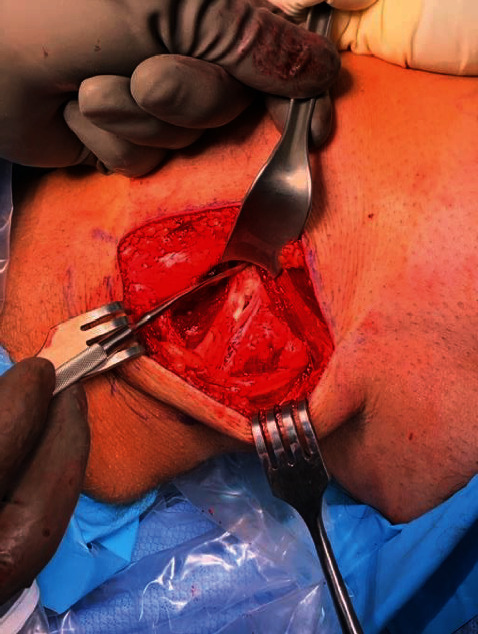
Lateral gastrocnemius tendon exposure.

**Figure 4:**
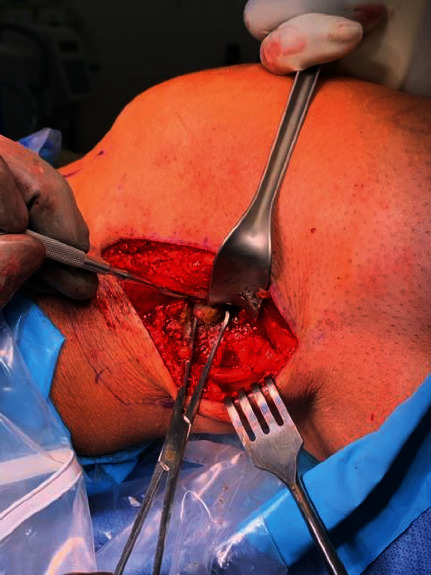
Fabella exposure with Allis forceps.

**Figure 5:**
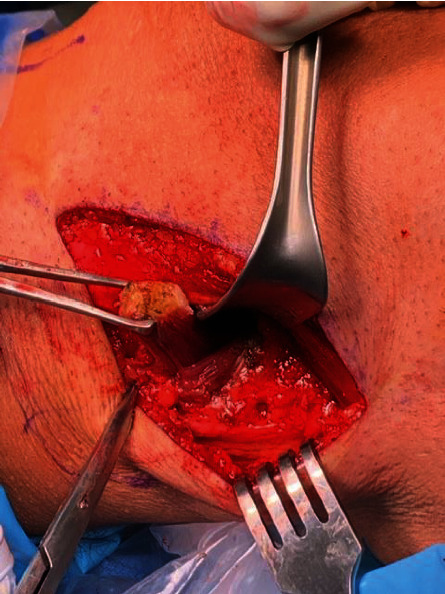
Fabella removal with Allis forceps.

**Figure 6:**
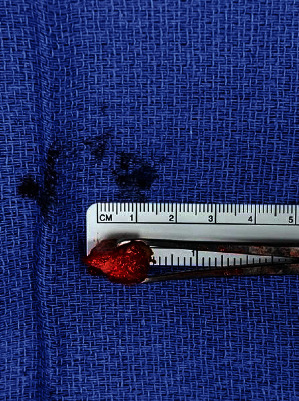
Fabella sizing (1.5 cm × 1 cm × 1 cm).

Next, the knee was brought through full range of motion to identify the snapping fabella in the popliteal fossa. The fabella was easily palpated in a knee range of motion. After careful dissection toward the capsular nodule, the fabella was identified within the lateral gastrocnemius tendon (Figure 3). Using a periosteal elevator and electrocautery, the 1.5 cm × 1 cm × 1 cm calcified lesion was exposed and then removed with Allies forceps (Figures 4–6). Moving the knee through a full range of motion again confirmed that snapping along the posterolateral knee was greatly reduced after fabella excision.

Postoperatively, the patient was allowed to bear weight as tolerated with use of a locked knee brace. He started a 6-week physical therapy program 2 weeks after the operation.

## 4. Outcome

Two weeks after fabella excision, the patient denied left knee pain, snapping, and associated radiating pain to his left. He noted minimal discomfort at the base of his left great toe and resolution of pain on the dorsal aspect of his foot. At 2 months, the patient reported minimal clicking with ambulation but denied lateral knee pain. His left knee range of motion was 0° to 130°. No tenderness to palpation along posterolateral origin of the gastrocnemius was appreciated. He was cleared to return to work 2 months after fabella excision.

## 5. Discussions

Fabella pathology is rare but increasingly recognized as a source of posterolateral knee pain following TKA. Initially believed to be unique to adolescents, fabella syndrome also affects older adults [5, 9]. When symptomatic, the fabella causes pain through mechanical irritation on the lateral femoral condyle, prosthetic implant, or soft tissues surrounding the lateral gastrocnemius tendon origin. Fabella pathology may present as earlier as the perioperative period, while also surfacing up to 9 years after TKA [10, 11].

The common peroneal nerve is vulnerable to compression at the knee [12]. Given its proximity to the lateral gastrocnemius tendon, the fabella may directly impinge the common peroneal nerve or inflame the surrounding soft tissues. Kimura et al. described a case of fabella-induced common peroneal nerve impingement producing extensor hallux weakness 8 years after TKA [13]. The authors suggested fabella enlargement and osteophyte formation as a possible explanation for delayed onset. Shen et al. reported a case of common peroneal nerve palsy produced by a fabella, which was recognized by weak great toe dorsiflexion and hyposensation over the superolateral foot 3 hours after TKA [11]. The patient's symptoms progressed until operative fabella excision 4 days following TKA. The significant size of the fabella and corrected contracture fracture were considered as contributing factors. Interestingly, neither of these patients presented with classic fabella snapping at the posterolateral knee. The present case is unique in that the patient reported common peroneal neuropathy symptoms along with classic fabella snapping at the posterolateral knee. Interestingly, the patient's work-up included electromyography which showed no abnormalities in the common peroneal nerve. The implication of the bifid nerve structure appreciated intraoperatively remains unknown.

The implication of TKA on fabella pathology is likely multifactorial due the anatomic complexity of the knee and significant changes associated with arthroplasty. The fabella has been reported to fracture following TKA, found incidentally in two cases, while three patients developed associated pain [10, 14]. Fabella fracture was hypothesized to be caused by preoperative valgus malignment or post-TKA varus alignment in these cases. Fabella size and shape are important considerations, as most reported cases involve a fabella over 1 cm. New biomechanics following TKA may stimulate fabella growth and osteophyte formation. Significant mechanical alignment changes may create eccentric mechanical loads across the gastrocnemius tendon and embedded fabella. Furthermore, the correction of a flexion contracture with TKA may affect soft tissues posterior to the knee including the gastrocnemius tendon.
